# Fundamental Movement Skills in Children and Adolescents—Bridging the Gap Between Theory and Practice

**DOI:** 10.3390/children13060786

**Published:** 2026-06-05

**Authors:** Jakub S. Gąsior

**Affiliations:** Department of Pediatric Cardiology and General Pediatrics, Medical University of Warsaw, 02-091 Warsaw, Poland; jakub.gasior@wum.edu.pl

## 1. Persistent Gaps and the Pediatric Inactivity Triad

Fundamental movement skills (FMSs), typically categorized into locomotor, object control, and stability skills, constitute the essential building blocks for more complex physical activities and are essential for participation in everyday tasks, sports, and physical activities [1,2,3]. These skills do not emerge naturally but instead require refinement through appropriate teaching and practice to achieve efficient form [4]. Over the last decade, there has been a dramatic increase in research on FMS, yet significant scientific gaps persist. This Special Issue of Children brings together six papers—including five original research articles and one comprehensive scoping review—addressing assessment tools, the relationship between FMSs and physical literacy, and the effectiveness of targeted interventions [5,6,7,8,9,10].

A cornerstone of this Special Issue is the scoping review by Piotrowski et al. [7], which highlights an alarming global decline in FMS acquisition [11,12,13]. This decline is particularly concerning when framed within the Pediatric Inactivity Triad [14,15], a clinical framework describing three interconnected conditions: physical illiteracy, exercise deficit disorder, and pediatric dynapenia:

- Physical Illiteracy—Insufficient motor-skill competence, confidence, or motivation to participate effectively in activities involving movement.
- Exercise Deficit Disorder: Failure to accumulate the recommended 60 min of moderate-to-vigorous physical activity (MVPA) daily.
- Pediatric Dynapenia: Measurably low muscular strength and power relative to age-appropriate norms.

The review by Piotrowski et al. emphasizes that FMSs should be interpreted as a vital component of physical literacy to combat this triad, noting that children with poor skills often struggle to break through a ‘motor proficiency barrier’ that predisposes them to hypoactivity and obesity. Furthermore, the review identifies several persistent scientific gaps: terminological confusion regarding motor competence and physical literacy; the absence of a universally accepted ‘gold standard’ assessment suitable for schools; the historical neglect of genetic and shared environmental factors; and the lack of an established algorithmic approach to determining the optimal intervention ‘dosage’ [7].

## 2. Advancing FMS Assessment and Reciprocal Relationships

In response to the need for refined diagnostic tools, Madu et al. investigated the factor structure of the Test of Gross Motor Development-2 [8]. By comparing equal- and differential-weighted factor structures, they concluded that while equal weighting is sufficient for general clinical use, differential weighting provides a more nuanced description of how individual skills relate to physical activity (PA) outcomes. This research supports the call for the development of more sophisticated diagnostic approaches to identify physical illiteracy early in childhood. This Special Issue also explores the reciprocal nature of FMSs and health. Madu et al. identified a positive feedback loop specifically between object control skills and MVPA during guided active play, aligning with models of integrated developmental trajectories [5]. Furthermore, Ltifi et al. found that among preschoolers, Body Mass Index is primarily associated with physical growth rather than immediate deficits in motor skills or executive function [9]. This finding suggests that the early years provide a critical ‘window of opportunity’ for universal interventions to promote healthy growth and combat the onset of the Pediatric Inactivity Triad before weight-related disparities in performance become entrenched.

## 3. Targeted Interventions for Holistic Growth

Structured interventions have demonstrated significant success in improving both physical and cognitive outcomes. Ltifi et al. showed that a 12-week mini-trampoline program significantly improved functional mobility, postural steadiness, and lower-body strength while also enhancing inhibitory control—a core component of executive function [6]. Similarly, Chiu et al. reported that an 8-week programmed physical activity intervention in Taiwan effectively boosted gross motor development, attention, and working memory [10]. These results reinforce the conclusion of the scoping review: FMS development is not a natural consequence of aging but requires structured, multidisciplinary opportunities for practice and teaching.

## 4. The Crucial Role of Multidisciplinary Collaboration in FMS Development

The roles of pediatric physicians, physiotherapists, and occupational therapists are paramount, as children with delayed psychomotor development [16], born prematurely [17], or affected by various pediatric conditions [18,19,20] all demonstrate significant delays in acquiring or deficits in executing FMSs. To meet the objectives of the 2030 Sustainable Development Agenda for high-quality early-childhood development, pediatric physicians must remain vigilant regarding widespread hypoactivity [21]. By using brief, validated screening tools such as the Physical Activity Vital Sign during routine clinical visits, they can identify insufficient activity levels and refer patients to appropriate specialists for further assessment [22]. Furthermore, physiotherapists and occupational therapists providing care for pediatric patients should routinely assess FMSs when formulating therapeutic goals under the “F-words” paradigm, which integrates fitness (structure/function), functioning (activity), and friends (participation) with environmental (family) and individual (fun) factors. Addressing the interconnected web of muscular strength (force), quality of experience (fun), and parental role models (family) is essential for children to break through motor proficiency barriers and establish lasting healthy habits [23,24].

## 5. Conclusions

The works collected in this Special Issue underscore that addressing the global decline in FMSs requires collaboration between teachers, parents, and healthcare providers. Future research must focus on standardizing assessment tools and establishing the optimal ‘dose’ of intervention to effectively combat the Pediatric Inactivity Triad and ensure a trajectory of lifelong health.
